# Differential contributions of ClpX and ClpP to pulmonary virulence in classical and hypervirulent *Klebsiella pneumoniae*

**DOI:** 10.1128/iai.00680-25

**Published:** 2026-01-30

**Authors:** Nathan M. Lin, Emily C. Marino, Jordan M. Schlotmann, David A. Rosen

**Affiliations:** 1 Division of Infectious Diseases, Department of Pediatrics, Washington University School of Medicine 12275 , St. Louis, Missouri, USA; 2 Department of Molecular Microbiology, Washington University School of Medicine 12275 , St. Louis, Missouri, USA; University of California San Diego School of Medicine, La Jolla, California, USA

**Keywords:** *Klebsiella pneumoniae*, virulence factors, pneumonia, Clp protease, antibiotic resistance

## Abstract

*Klebsiella pneumoniae* is an opportunistic Gram-negative pathogen and a common cause of antibiotic-resistant infections including neonatal sepsis and hospital-acquired pneumonia. *K. pneumoniae* strains can be categorized into two pathotypes: classical *K. pneumoniae* (cKp), which often causes nosocomial infections, and hypervirulent *K. pneumoniae* (hvKp), which can cause severe disease in healthy hosts. New therapies are urgently needed for these infections, and caseinolytic proteins have emerged as promising therapeutic targets in other bacterial pathogens. ClpX and ClpP have been implicated in bacterial protein homeostasis, regulation of virulence, and antimicrobial susceptibility in other species, but their specific roles in *K. pneumoniae* pathogenesis have yet to be defined. Here, we investigate the contribution of *K. pneumoniae* ClpX and ClpP to lung infection, virulence factor regulation, and antibiotic susceptibility. In a murine pneumonia model, loss of ClpX impairs infection of both hvKp and cKp. Loss of ClpX results in decreased capsule production in hvKp and enhances type 1 pilus production in both pathotypes. In hvKp, loss of ClpX increases type 3 pili, while in cKp, increased type 3 piliation is observed with loss of ClpP. Across both pathotypes, loss of ClpX or ClpP increases susceptibility to a range of antibiotics. These data identify ClpX as critical to *K. pneumoniae* virulence and antimicrobial susceptibility. By connecting ClpX to capsule production, pili regulation, and *in vivo* virulence, this work highlights a conserved putative therapeutic target that may enable adjunctive strategies to enhance antibiotic efficacy or attenuate the severity of *K. pneumoniae* infection.

**IMPORTANCE:**

*Klebsiella pneumoniae* is a leading cause of antibiotic-resistant and hospital-acquired infections. The emergence of highly virulent strains of *K. pneumoniae* capable of causing severe disease is of utmost concern. Here, we investigate two specific caseinolytic proteins, ClpX and ClpP, produced by both classical and hypervirulent strains of *K. pneumoniae* and their role in *K. pneumoniae* lung infection. We show that ClpX is a key regulator of virulence factors including bacterial pili and capsule; it is essential for murine pulmonary fitness across both classical and hypervirulent pathotypes. Furthermore, loss of ClpX increases susceptibility to multiple antibiotics, indicating a role in both protein homeostasis and pathogenicity. These findings suggest ClpX is a conserved virulence determinant in multiple strains of *K. pneumoniae* and highlight its potential as a therapeutic target to enhance antibiotic efficacy or mitigate disease severity.

## INTRODUCTION

*Klebsiella pneumoniae* is a Gram-negative, facultative anaerobe and member of the order Enterobacterales. This opportunistic human pathogen commonly causes nosocomial infections such as pneumonia, sepsis, and urinary tract infections. In recent years, the Centers for Disease Control and Prevention (CDC) identified *K. pneumoniae* as an urgent public health threat due to its rapidly increasing antibiotic resistance (1). The Child Health and Mortality Prevention Surveillance (CHAMPS) Network identified *K. pneumoniae* as a leading cause of neonatal sepsis and the most common etiological agent in infectious deaths in children under the age of five (2). Historically, the majority of *K. pneumoniae* infections were caused by classical *K. pneumoniae* (cKp) strains that infected hospitalized patients, neonates, and hosts with compromised immune systems. In recent decades, strains of hypervirulent *K. pneumoniae* (hvKp) have emerged that are capable of infecting healthy hosts and causing severe infection (3). Furthermore, multi-directional genetic exchanges between cKp and hvKp have resulted in multidrug-resistant strains of hvKp (4, 5).

A transposon-mutant library screen in a hypervirulent strain of *K. pneumoniae* was used to identify genes needed for bacterial fitness in the lung (6). This study identified several previously known and unknown putative virulence factors, some of which were corroborated using mutant strains in murine models. The genes *clpX* and *clpP* were identified as putative fitness factors in the murine lung but were not investigated further in pathogenesis experiments. Genomically, *clpP* and *clpX* form a transcriptional unit with *clpX* immediately downstream of *clpP* (7); despite this orientation, the majority of the literature refers to this transcriptional unit and the protein complex as *clpXP* and ClpXP, respectively. ClpX and ClpP are caseinolytic proteins (Clp) essential for protein homeostasis and quality control in other bacteria (8). ClpX is a highly conserved hexameric AAA+ ATPase unfoldase/chaperone (9–11). ClpP is a critical serine peptidase that is active as a multimer (12). While both proteins have independent functions, they also can form the ClpXP complex—an ATP-dependent molecular machine that unfolds and degrades proteins containing specific tags (12). Furthermore, ClpX and ClpP are highly conserved within both the *Klebsiella* genus and other prokaryotic organisms (9, 13). Despite characterization in other pathogens (including *Staphylococcus aureus, Streptococcus pneumoniae,* and *Escherichia coli*), ClpX, ClpP, and the ClpXP complex have yet to be fully investigated in *K. pneumoniae* (14–17). Broadly, ClpX, ClpP, and the ClpXP complex have been shown in other bacterial species to impact overall stress response, antimicrobial resistance, and the production of virulence factors (18–20). In *Bacillus anthracis* and *S. aureus,* inhibition of ClpX or ClpP has been shown to sensitize these pathogenic bacteria to antimicrobial agents (21, 22). Recent studies have also identified Clp proteins as promising targets for novel therapeutics in a range of bacteria from *Mycobacterium tuberculosis* to *S. aureus (*23–25).

Given the global prevalence of *K. pneumoniae* and its high rates of multidrug resistance, studies investigating its mechanisms of virulence are needed to inform the development of novel therapeutics. The conservation of ClpX and ClpP across bacterial organisms, integration in virulence pathways, and emerging status as drug targets underscore an unmet need to better understand the role of these proteins in *K. pneumoniae* pathogenesis. The goal of this study was to investigate the role of ClpX and ClpP in the virulence of both cKp and hvKp. We generated isogenic knockouts of *clpX, clpP,* and *clpXP* in cKp strain TOP52 and hvKp strain ATCC 43816 and characterized virulence-associated phenotypes *in vitro* and in a murine pneumonia model.

## RESULTS

### Loss of ClpX or ClpP increases susceptibility of *K. pneumoniae* to multiple classes of antibiotics

To evaluate the role of ClpX and ClpP in both cKp and hvKp, we constructed isogenic knockouts in TOP52 (cKp) and 43816 (hvKp) resulting in strains TOP52*∆clpX,* TOP52*∆clpP,* TOP52*∆clpXP*, 43816*∆clpX,* 43816*∆clpP,* and 43816*∆clpXP*. Both TOP52 and 43816 demonstrated similar growth kinetics over 18 h to their respective Clp mutants (Fig. S1). As others have demonstrated that *clpXP* influences antimicrobial susceptibility of *S. aureus, B. anthracis,* and *E. coli*, we sought to determine if these genes similarly affect *K. pneumoniae* antimicrobial susceptibility (21, 22, 24, 26). We quantified the minimum inhibitory concentration of four antimicrobial agents against the wild-type and mutant strains. Loss of either ClpX or ClpP increased susceptibility to kanamycin, gentamicin, and chloramphenicol in both pathotypes (Table 1).

**TABLE 1:** Antibiotic minimum inhibitory concentrations for wild-type and mutant strains

Antimicrobial	MIC (µg/mL)				MIC (µg/mL)			
	43816^*a*^	43816*∆clpX*	43816*∆clpP*	43816*∆clpXP*	TOP52^*a*^	TOP52*∆clpX*	TOP52*∆clpP*	TOP52*∆clpXP*
Kanamycin	**8**	4	4	4	**8**	4	2	1
Tetracycline	**1**	1	1	0.5	**1**	1	0.25	0.25
Gentamicin	**2**	1	1	1	**2**	1	0.5	0.5
Chloramphenicol	**16**	4	4	2	**4**	2	1	2

^*a*^ Wild-type MICs are highlighted in bold text.

### The loss of ClpX attenuates capsule production in hvKp but not cKp

**Fig 1:**
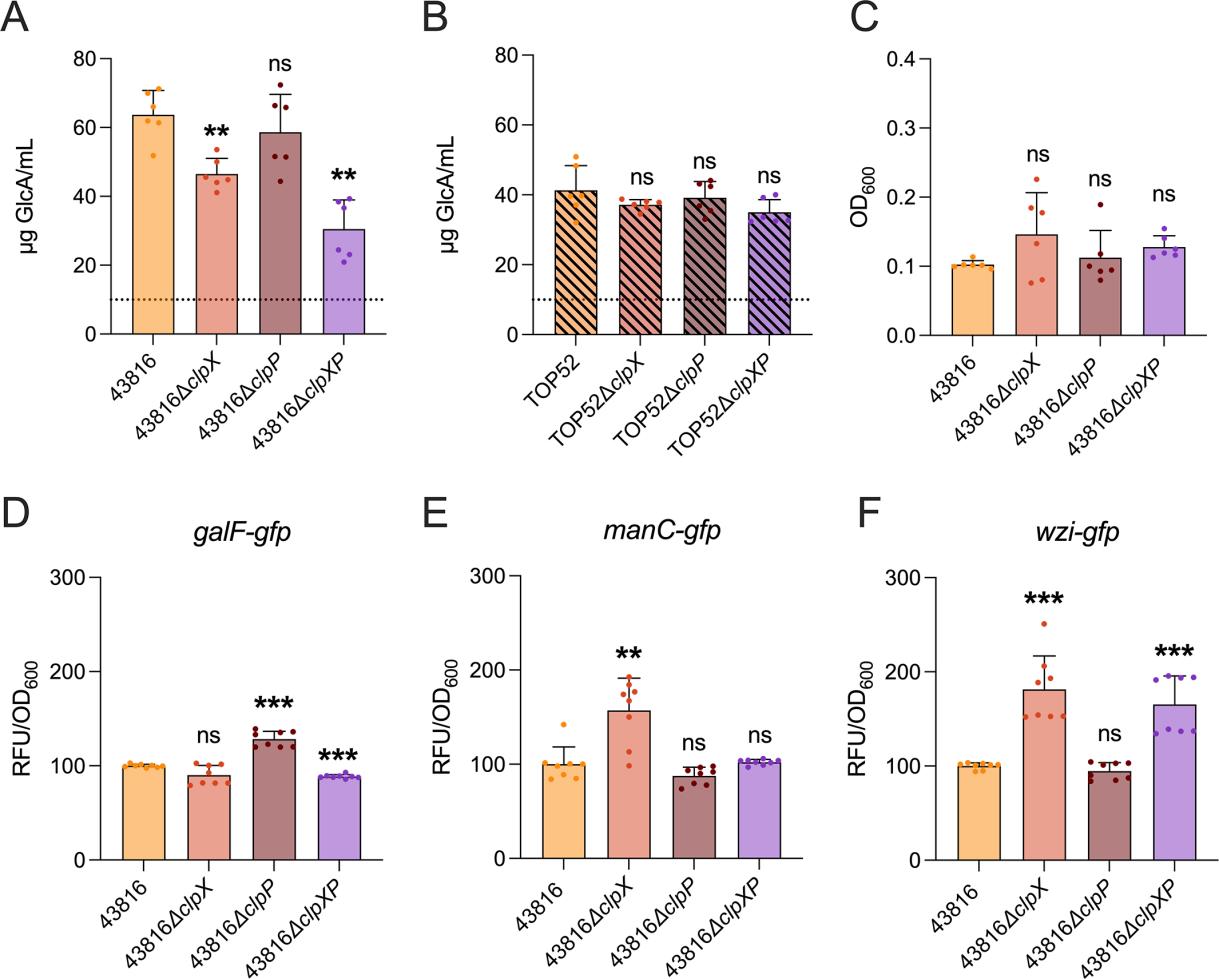
Loss of ClpX reduced uronic acid in 43816 (**A**) but not in TOP52 (**B**). The loss of ClpX or ClpP does not alter the mucoviscosity of 43816 (**C**). The expression of capsular promoter gene, *galF* (**D**), is influenced by ClpX and ClpP while *wzi* (**E**) and *manC* (**F**) are influenced by ClpX. Relative fluorescence units (RFU) were measured, normalized to the culture OD_600_, and set relative to the wild-type RFU. The data in panels **A–C** were compiled from two independent experiments with samples analyzed in triplicate. Significance was determined through comparison to the wild-type background (**P < 0.01 by the Mann-Whitney *U* test with Holm-Šidák correction). The data panels **D–F** were compiled from two independent experiments with samples done in quadruplicate. Significance was determined through comparison to the wild-type background (**P < 0.01, ***P < 0.001 by the Mann-Whitney *U* test with Holm-Šidák correction). ns, not significant.

Capsule, an extracellular polysaccharide matrix that promotes immune evasion, is a critical determinant of pulmonary virulence and is typically produced in abundance by hvKp isolates (3). To determine if ClpX or ClpP influences capsule production in *K. pneumoniae*, we quantified uronic acid—a major component of *K. pneumoniae* capsule (Fig. 1A and B). Wild-type 43816 exhibited greater capsule production than 43816∆*clpX* or 43816∆*clpXP*, but not 43816∆*clpP*. TOP52∆*clpX*, TOP52∆*clpP,* and TOP52∆*clpXP* all had similar levels of capsule production compared to wild-type TOP52. These data suggest that loss of ClpX, but not ClpP, decreases capsule production in hvKp but not in cKp.

Capsule is often correlated with mucoviscosity in hvKp. To evaluate if reductions in capsule altered the mucoviscosity of 43816, a low-speed centrifugation assay was conducted to determine the level of mucoviscosity (Fig. 1C). Compared to wild-type 43816, there were no changes to mucoviscosity in 43816*∆clpX,* 43816*∆clpP*, or 43816*∆clpXP*.

The production of capsule is a complex process requiring the expression of numerous genes organized within the capsule locus (*cps*). There are three promoters within the *cps* locus of K2 strains (such as 43816) that are immediately upstream of genes *galF*, *wzi*, and *manC* (27). To identify Clp-dependent alterations in transcriptional regulation of capsule in 43816, we utilized *gfp* reporter fusions to the *galF, wzi,* and *manC* promoters, transformed into wild-type 43816, 43816∆*clpX,* 43816∆*clpP*, and 43816∆*clpXP* (Fig. 1D through F). The expression of *galF* was lower in 43816*∆clpXP* compared to wild-type 43816 but higher in 43816*∆clpP*. Wild-type 43816 had lower expression of *wzi* than 43816*∆clpX* or 43816*∆clpXP*, but not 43816*∆clpP*. Compared to wild-type 43816, the expression of *manC* was higher in 43816*∆clpX* but not significantly different in 43816*∆clpP* or 43816*∆clpXP*. These data suggest that loss of ClpX in 43816 increases *wzi* and *manC* expression and decreases *galF* expression, while loss of ClpP increases *galF* expression. A reduction in *galF* expression is consistent with the attenuated capsule production observed in 43816*∆clpXP* and suggests ClpX-based regulation of *galF*.

### Clp protease mutants minimally affect epithelial cell interactions *in vitro*

**Fig 2:**
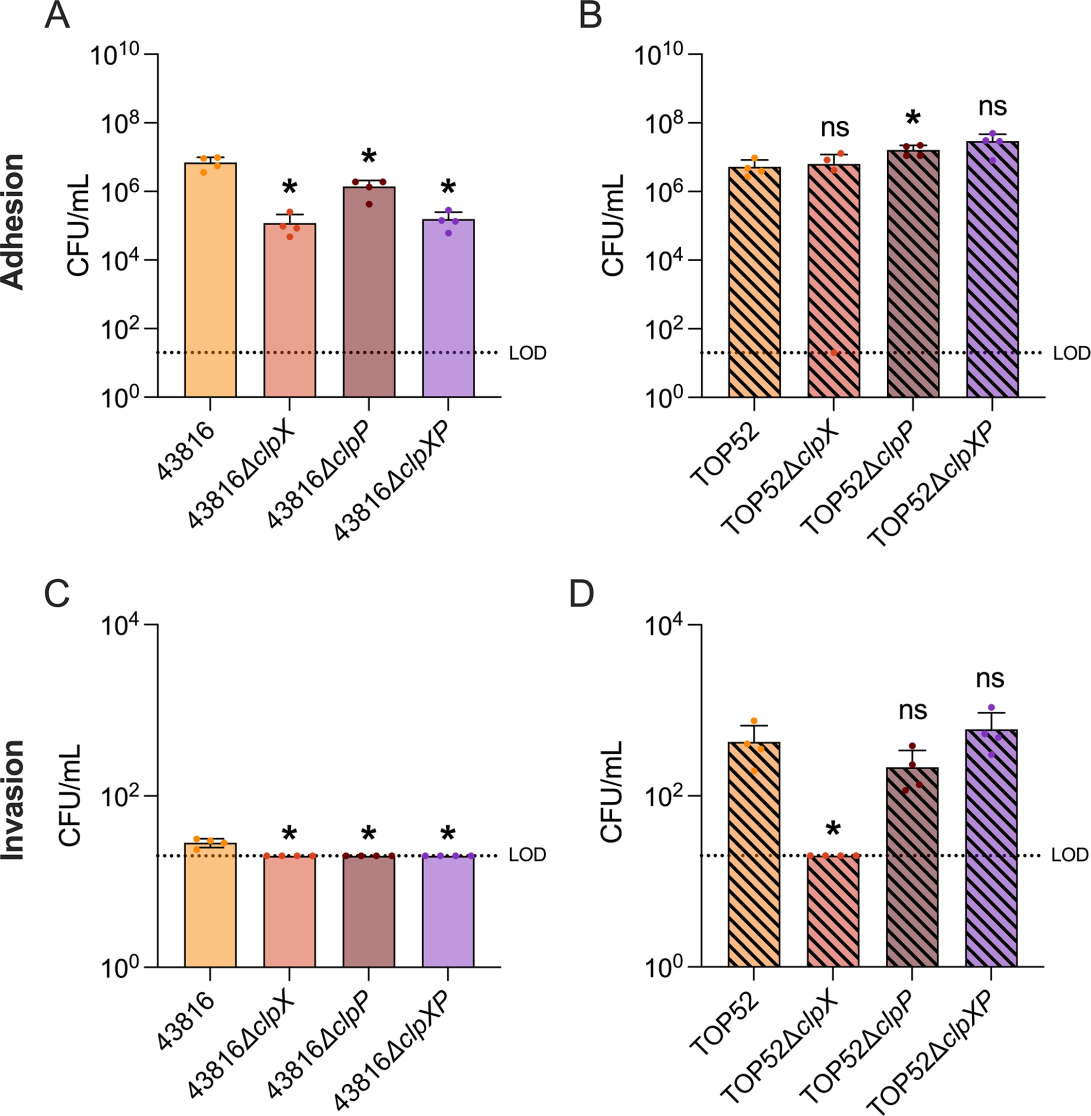
Clp protease mutants minimally alter epithelial adhesion and invasion. The loss of ClpX or ClpP reduces adhesion in 43816 (**A**). The loss of ClpP increases adhesion in TOP52 (**B**). The loss of ClpX or ClpP reduces invasion in 43816 (**C**). The loss of ClpX reduces invasion in TOP52 (**D**). The data in panels **A–D** are a representative assay. All samples were done in quadruplicate with a MOI of 10 and normalized to MTT OD_570_. Significance was determined through comparison to the wild-type background (*P < 0.05, by the Mann-Whitney *U* test with Holm-Šidák correction). ns, not significant.

Adhesion to a mucosal surface is often a necessary step in establishment of a successful infection (28). Adhesion to airway epithelia facilitates early colonization and may promote internalization into nonphagocytic cells (28, 29). To elucidate if ClpX or ClpP influences hvKp or cKp interactions with lung epithelial cells, we quantified *K. pneumoniae* binding and internalization with A549 cells (Fig. 2). Wild-type 43816 exhibited greater adhesion to and invasion into A549 cells than 43816*∆clpX,* 43816*∆clpP*, and 43816*∆clpXP*. Meanwhile, TOP52*∆clpP,* but not TOP52*∆clpX* or TOP52*∆clpXP*, had modestly greater adhesion than wild-type TOP52; only TOP52*∆clpX* had lowered invasion. These data suggest that ClpX and ClpP exert small but significant impacts on hvKp and cKp adhesion and invasion of lung epithelial cells; however, these differences may or may not be physiologically meaningful in the context of overall virulence.

### ClpX and ClpP differentially impact abundance of type 1 and type 3 pili across *K. pneumoniae* pathotypes

**Fig 3:**
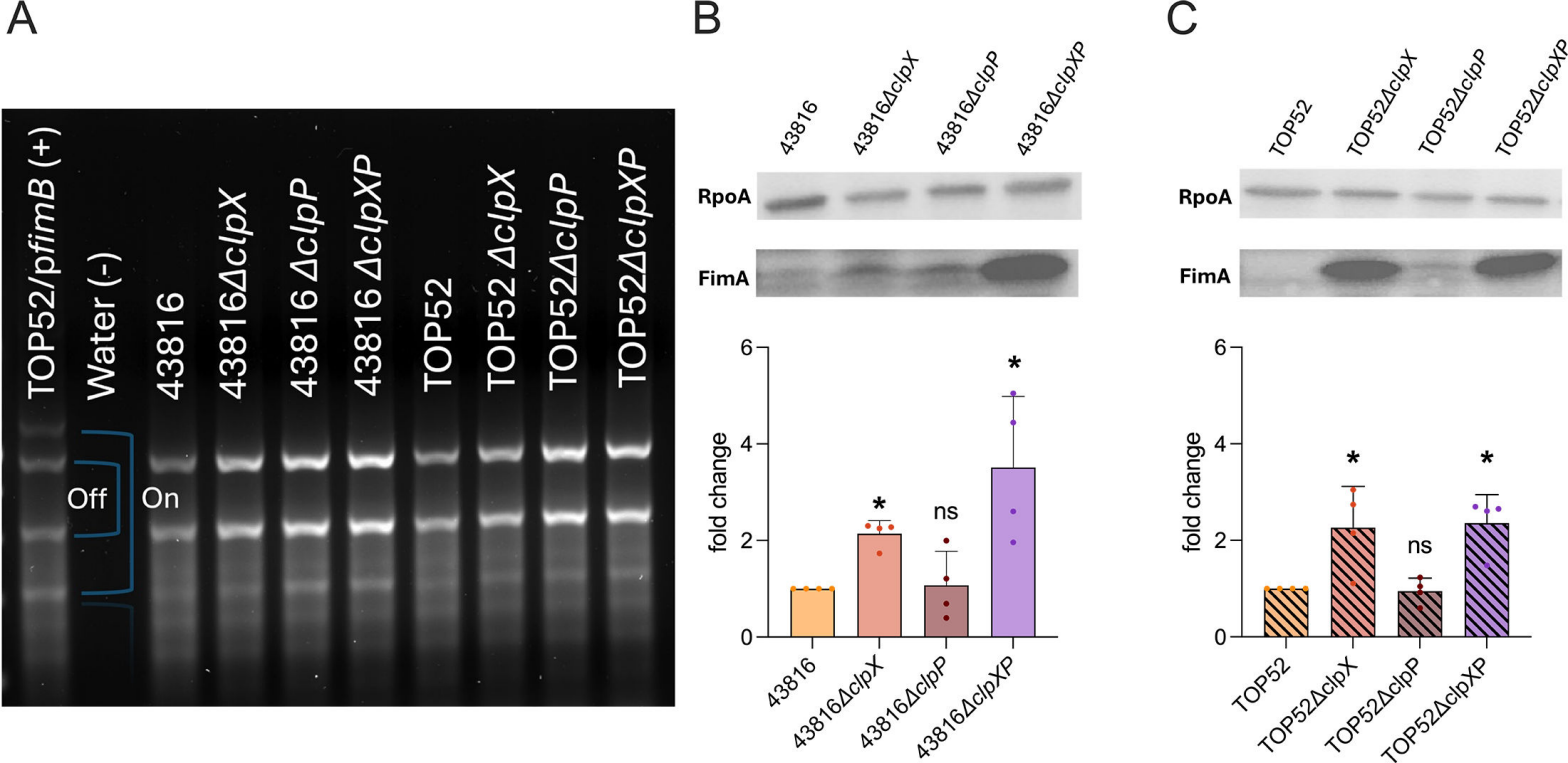
ClpX and ClpP do not impact *fimS* control of type 1 pili but the loss of ClpX leads to overexpression of FimA in TOP52 and 43816. A standard PCR with primers phase A and phase B was conducted with gDNA from WT and mutant strains of 43816 and TOP52 to amplify *fimS* (**A**). Immunoblotting of FimA and the control protein RpoA in 43816 wild-type and mutant strains (**B**) and TOP52 wild-type and mutant strains (**C**) to determine type 1 pili expression. Significance was determined through comparison to the wild-type background (*P < 0.05 by the Mann-Whitney *U* test with Holm-Šidák correction). ns, not significant.

Previous studies have implicated *E. coli* ClpXP in the regulation of type 1 pili, which mediate bacterial adhesion during urinary tract infection*,* but this effect has yet to be studied in *K. pneumoniae* (17). To evaluate if ClpX or ClpP mediates type 1 pili expression, we examined the orientation of the invertible DNA promoter element *fimS* using a PCR-based assay (Fig. 3A) (30). *fimS* is the primary transcriptional regulator of type 1 pili in *K. pneumoniae* and can be in one of two positions: ON or OFF (31). *fimB* is a recombinase that favors the ON position of *fimS*, and a copy of *fimB in trans* was utilized as a positive control. Visualization of amplified *fimS* revealed it to be predominantly OFF in wild-type 43816 and TOP52. There was no observed change in the *∆clpX, ∆clpP,* or *∆clpXP* mutants in either background strain. These results indicate that neither ClpX nor ClpP significantly alters the orientation of the *fimS* switch at this timepoint, which remains primarily OFF in *K. pneumoniae*.

To determine if ClpX or ClpP influences production of type 1 pili, we performed immunoblots for FimA, the primary structural subunit of type 1 pili (Fig. 3B and C). 43816*∆clpX* and 43816*∆clpXP,* but not 43816*∆clpP,* had higher abundance of FimA compared to wild-type 43816. Similarly, TOP52*∆clpX* and TOP52*∆clpXP* had higher abundance of FimA, while TOP52*∆clpP* showed no change compared to wild-type TOP52. The results of these immunoblots suggest that ClpX has a conserved role in decreasing the production of type 1 pili in both hvKp and cKp.

**Fig 4:**
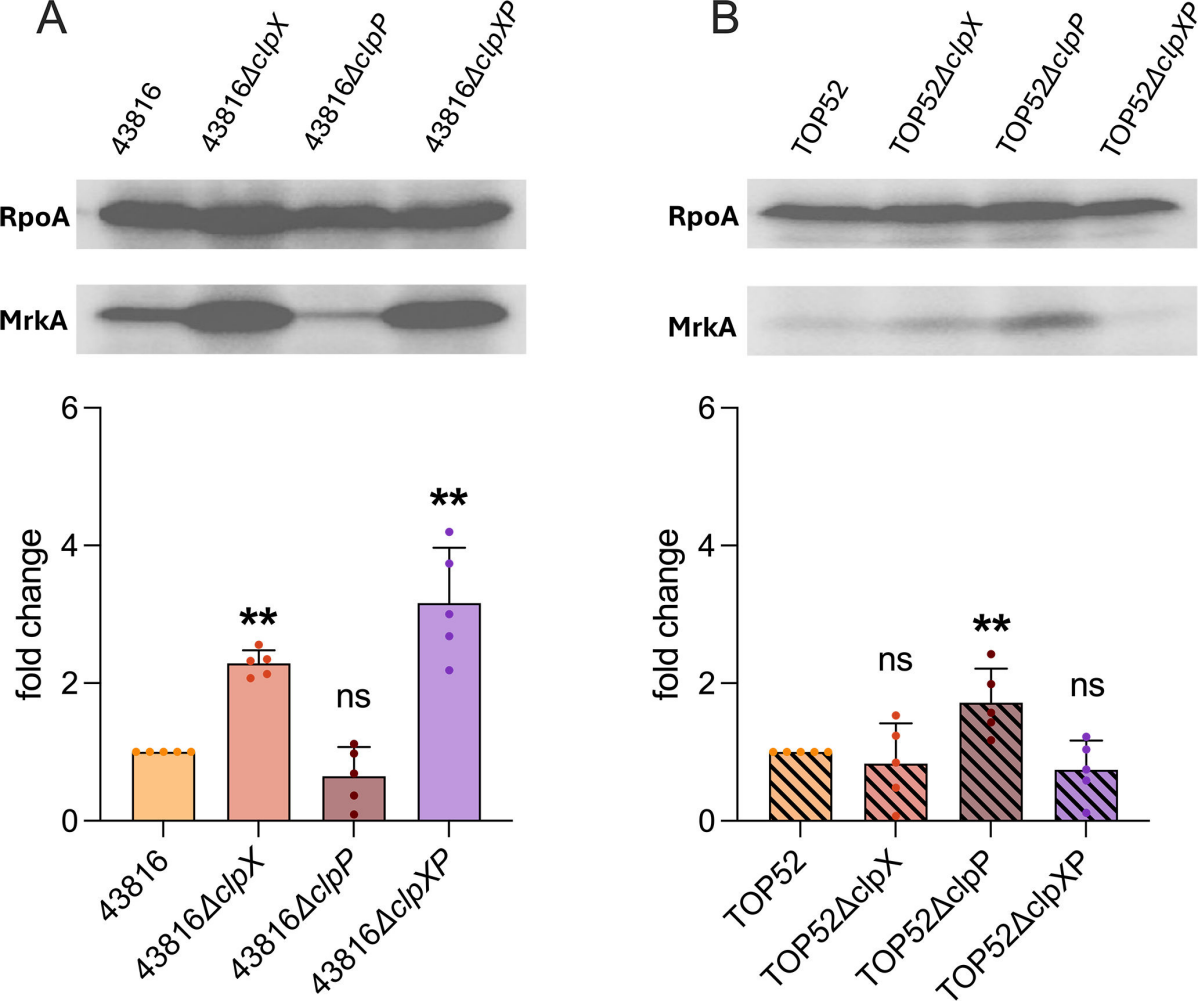
ClpX and ClpP influence type 3 pili in a pathotype-dependent manner. Immunoblotting of MrkA and the control protein RpoA in 43816 wild-type and mutant strains (**A**) and TOP52 wild-type and mutant strains (**B**) to determine type 3 pili expression. Significance was determined through comparison to the wild-type background (**P < 0.01 by the Mann-Whitney *U* test with Holm-Šidák correction). ns, not significant.

Another adhesive fimbrial structure, type 3 pili, promotes biofilm formation and adherence of *K. pneumoniae* to abiotic surfaces (32–35). To characterize type 3 pili expression, we immunoblotted for MrkA, the primary structural subunit of type 3 pili (Fig. 4A and B). 43816*∆clpX* and 43816*∆clpXP* had higher MrkA abundance than wild-type 43816, while 43816*∆clpP* had no change. TOP52*∆clpX* and TOP52*∆clpXP* had no change in MrkA levels, while TOP52*∆clpP* had higher levels of MrkA compared to wild-type TOP52. These data suggest ClpX is involved in the regulation of type 3 pili in hvKp but not cKp. Conversely, ClpP appears to be important in the regulation of type 3 pili in cKp but not hvKp. Together, this indicates that ClpX and ClpP influence type 3 pili in *K. pneumoniae* in a pathotype-specific manner.

### ClpX is required for hvKp and cKp lung pathogenesis and dissemination

**Fig 5:**
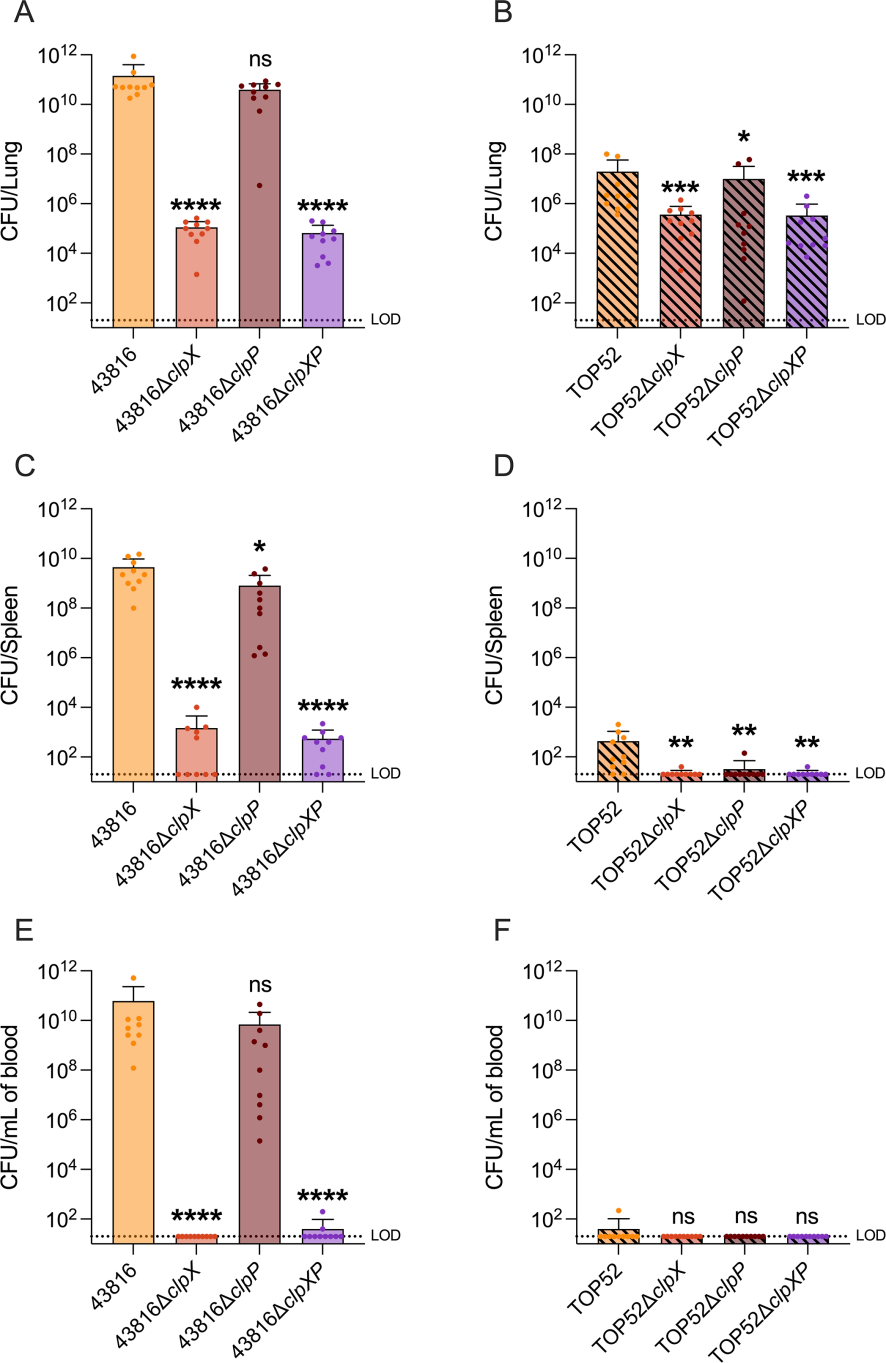
The loss of ClpX reduces virulence of TOP52 and 43816 while the loss of ClpP only reduced TOP52 virulence in a murine model of pneumonia. Mice were inoculated with between 10^8^ and 10^9^ CFU of selected strains and sacrificed 24 hpi. Lungs, spleens, and blood were removed. The organs were homogenized and collected samples were plated to determine bacterial burden. The data for 43816 (**A–C**) and TOP52 (**D–F**) were compiled from two independent experiments. The limit of detection was 20 CFU. Significance was determined through comparison to the wild-type background (*P < 0.05 and **P < 0.01, ***P < 0.001, and ****P < 0.0001, by the Mann-Whitney *U* test with Holm-Šidák correction). ns, not significant; LOD, limit of detection.

Successful pathogenesis in a host is a complex, multi-faceted process. To determine the role of ClpX and ClpP in *K. pneumoniae* pathogenesis, we utilized a murine model of pneumonia (Fig. 5). Wild-type female CD-1 mice were inoculated by oropharyngeal aspiration with 43816 or TOP52 or Clp mutants (*∆clpX, ∆clpP,* or *∆clpXP*). At 24 h post-infection (hpi), we collected lungs, spleen, and blood to quantify bacterial burden. Wild-type 43816 established greater lung bacterial burden than 43816∆*clpX* or 43816∆*clpXP*, but not 43816∆*clpP*. TOP52*∆clpX,* TOP52*∆clpP*, and TOP52*∆clpXP* all had reduced lung bacterial burden compared to wild-type TOP52, with the most significant reductions in TOP52*∆clpX* and TOP52*∆clpXP*. In the spleen, 43816∆*clpX* and 43816∆*clpXP* had significantly reduced bacterial burden, while 43816∆*clpP* had slightly lower bacterial burden than 43816. Wild-type TOP52 had higher bacterial burden in the spleen than TOP52*∆clpX*, TOP52*∆clpP*, or TOP52*∆clpXP*. In the blood, wild-type 43816 established higher bacterial burden than 43816*∆clpX* and 43816*∆clpXP*, but not 43816*∆clpP*. TOP52∆*clpX*, TOP52∆*clpP,* and TOP52*∆clpXP* all had similarly low bacterial burden in the blood compared to wild-type TOP52. These data suggest that, while ClpP may exert a small role in *K. pneumoniae* pathogenesis, ClpX is a critical fitness factor for both hvKp and cKp during lung infection.

## DISCUSSION

The rapid rise of drug resistance in *K. pneumoniae* and the increasing prevalence of hvKp isolates pose major challenges to infection management and global health. As the pipeline for novel antibiotics has slowed, there remains a significant need for new and effective therapeutics. Clp proteases have emerged as modulators of both virulence and antimicrobial susceptibility in several human pathogens. In the present work, we expand upon existing knowledge of these factors by characterizing ClpX and ClpP in *K. pneumoniae*.

We delineated distinct roles of ClpX and ClpP in hvKp and cKp virulence using a murine pneumonia model and *in vitro* assays. Loss of ClpX attenuated lung infection in both hvKp (43816) and cKp (TOP52) strains, whereas ClpP was generally dispensable in hvKp but had modest contributions to cKp pathogenesis. In both hvKp and cKp, the loss of ClpX reduced the bacterial burden in the blood and spleen. However, these data could reflect attenuated virulence at the primary site of infection rather than ClpX acting as an independent dissemination factor. Our *in vivo* data suggest shared dependence on ClpX for pulmonary virulence, with a divergent reliance on ClpP between pathotypes. ClpP might still contribute to hvKp virulence, albeit clearly to a lesser degree than in cKp; for example, in murine pneumonia, 43816*∆clpP* had reduced dissemination to the spleen 24 hpi. Furthermore, given the higher virulence of hvKp overall, small effects on virulence might be more difficult to detect. Clearly, the dramatic effect of ClpX loss, but not ClpP loss, suggests that the imperative role of ClpX in this host pulmonary niche is independent of the ClpXP complex.

The contributions of ClpX to pulmonary virulence are likely multifactorial; however, our experiments hint at two potential mechanistic explanations for the *in vivo* hvKp phenotype—namely, that ClpX loss reduces capsule abundance or increases piliation. Reduced capsule production may reflect decreased transcription of the *galF* promoter. GalF catalyzes the formation of UDP-d-glucose, an essential precursor for the synthesis of capsular polysaccharides (36). Without adequate quantities of this enzyme, capsule synthesis is limited. ClpX-based reduction of *galF* was observed in a previous study which identified that loss of ClpX in hvKp strain NTUH-K2044 led to reduced *galF* RNA levels (7). However, this is the first study to investigate changes to *wzi* and *manC* associated with ClpX or ClpP loss, which we hypothesize may be increased in a compensatory response to reduced *galF* activity. The increased expression of *wzi* and *manC* likely did not increase capsule levels due to the restricted upstream supply of UDP-d-glucose. Capsule is a widely established and well-studied virulence factor in *K. pneumoniae*. In the pulmonary environment, capsule is known to reduce host immunological responses, inhibit complement-mediated killing, and reduce susceptibility to phagocytosis (37–39). Given the multi-faceted role of capsule during pulmonary infection, the observed reduction in capsule provides a likely explanation for attenuated pulmonary virulence. However, further investigation is necessary to elucidate the mechanism by which ClpX influences *galF* activity in 43816 either directly or indirectly.

Loss of ClpX increased expression of both type 1 (FimA) and type 3 (MrkA) pili in *K. pneumoniae*, implying that ClpX normally constrains pili abundance. While we detected no differences in type 1 promoter orientation by phase assay, we demonstrated increased total FimA by immunoblot. This suggests that ClpX may influence *fimA* expression independent of phase variation at the given timepoint assessed or that the immunoblotting technique may be more sensitive for detecting differences in type 1 piliation. While the absence of FimA or MrkA alone has not been shown to alter virulence of *K. pneumoniae* in the lung, the overexpression of fimbriae may be detrimental to virulence (40). A previous study in cKp demonstrated that high c-di-GMP levels increased production of type 1 pili, but not type 3 pili, leading to attenuated virulence in the respiratory tract (41). Furthermore, fimbriae may be recognized by phagocytic cells, promoting bacterial killing in the immune cell-rich lung environment (42, 43). In hvKp, the overexpression of type 1 and type 3 pili in the ClpX knockout, in tandem with reduced capsule production, likely increases immune recognition and bacterial clearance in the lung.

In cKp, where baseline capsule production is significantly lower than hvKp, we were not able to detect significant changes in capsule with the loss of ClpX or ClpP. However, the prominent increase in pili may partially explain the virulence attenuation. Interestingly, we observed that ClpP is necessary for the regulation of type 3 pili, while ClpX is required for the regulation of type 1 pili. In our murine infection model, loss of either ClpX or ClpP reduced bacterial burden in the lung, with a more significant attenuation associated with ClpX. Significant reduction in dissemination from the lung is also observed with both ClpX and ClpP loss, though TOP52, like most cKp strains, has a low propensity for dissemination at baseline.

The differential phenotypes observed between the *clpX* and *clpP* mutants may also be influenced by additional proteases or adapter proteins. A closely related protease, ClpA, has been shown to form the ClpAP complex in some Enterobacteriales that exhibits a substrate specificity which partially overlaps that of ClpXP (44, 45). Our findings suggest that overlapping ClpAP activity does not meaningfully compensate for ClpX loss in *K. pneumoniae*. While other proteases such as Lon and FstH are capable of degrading abnormal proteins, these systems cannot replicate ClpX’s ClpP-independent chaperone-like functions (46, 47). Thus, the phenotype observed with ClpX loss may reflect impaired refolding of misfolded proteins rather than a major loss of proteolytic activity. The milder phenotype associated with loss of ClpP may be, in part, related to potential compensation by Lon, FstH, and the SsrA pathway for general protein degradation. Finally, several adaptor proteins such as SspB and RssB can provide substrate specificity to ClpXP (48–50). Although these adaptor proteins were not identified in the initial transposon screen, further investigation into their activity in the absence of ClpX or ClpP could further clarify the observed phenotypes. Future studies should better dissect the role of adaptor proteins for ClpXP in the context of *K. pneumoniae* virulence as well as evaluate compensatory changes to other proteases such as Lon with the loss of ClpX or ClpP.

Several factors limit the full scope of conclusions drawn from this study. While alterations to virulence factors such as capsule and pili were observed in *clpX* and *clpP* mutants, our experiments did not assess whether these effects were direct or indirect. Some published data suggest counter-regulation between certain pilus operons (such as type 3) and capsule (51). Additional investigation is necessary to clarify the exact mechanisms whereby ClpX influences capsule and pilus production in *K. pneumoniae*. Moreover, while the present work establishes initial investigation of ClpX and ClpP in the virulence of classical and hypervirulent *K. pneumoniae*, further investigation with additional isolates is required to generalize our findings to the broader pathotypes. Finally, complementation of the deleted genes could not be readily accomplished given the increased antibiotic susceptibility of each *clp* mutant and the need for positive antibiotic selection markers. All mutants, however, were fully sequenced to ensure no unexpected or off-target mutations.

The increased susceptibility of ∆*clpX* and ∆*clpP* mutants in both *K. pneumoniae* pathotypes to several antibiotics indicates that ClpX and ClpP may be necessary for stress tolerance or protein homeostasis to prevent lethal protein aggregation in the context of antibiotic stress. The conserved role of ClpX in regulating virulence attributes of cKp and hvKp, in tandem with the enhanced antimicrobial susceptibility associated with loss of ClpX, situates ClpX as a promising therapeutic target. Importantly, it demonstrates potential as monotherapy for acute *K. pneumoniae* infection or as adjunctive therapy to sensitize *K. pneumoniae* to existing agents. Currently, several promising ClpX inhibitors have been investigated in other bacterial pathogens (21, 52). Future studies utilizing these existing ClpX inhibitors in *K. pneumoniae* is a logical next step of translational investigation.

## MATERIALS AND METHODS

### Bacterial strains, reagents, and culture conditions

The *K. pneumoniae* hvKp strain American Type Culture Collection (ATCC) 43816, cKp strain TOP52, and mutants derived from these wild-type isolates were used for all experiments (Table 2). *K. pneumoniae* cultures were grown static at 37°C in Luria-Bertani (LB) broth unless otherwise specified. Growth medium was supplemented with 50 µg/mL of the appropriate antibiotic when necessary for positive selection. The bacterial strains and primers used in this study are described in Table 2 and Table S1, respectively. For preparation of murine inoculum, overnight cultures were centrifuged at 8,000 × *g* for 10 min and resuspended in sterile phosphate-buffered saline (PBS) to a standardized OD_600_. Murine inoculum was confirmed through serial dilution and plating on LB agar. All primers used were designed using SnapGene and sourced from IDT.

**TABLE 2:** Bacterial strains and plasmids used in this study

Strain	Description	Reference
43816	Hypervirulent *K. pneumoniae* isolate	(53)
TOP52	Classical *K. pneumoniae* isolate	(54)
43816∆*clpX*	Knockout of *clpX* in 43816	This study
43816∆*clpP*	Knockout of *clpP* in 43816	This study
43816∆*clpXP*	Knockout of *clpXP* in 43816	This study
TOP52∆*clpX*	Knockout of *clpX* in TOP52	This study
TOP52∆*clpP*	Knockout of *clpP* in TOP52	This study
TOP52∆*clpXP*	Knockout of *clpXP* in TOP52	This study
p*fimB*	*fimB* cloned into pBAD33; P_ara_ Cm^R^	This study
pPROBE-*manC*	GFP transcriptional reporter promoter fusion, *manC* promoter; Kan^R^	(55)
pPROBE-*galF*	GFP transcriptional reporter promoter fusion, *galF* promoter; Kan^R^	(55)
pPROBE-*wzi*	GFP transcriptional reporter promoter fusion, *wzi* promoter; Kan^R^	(55)

### Construction of gene knockouts and plasmids

Lambda Red recombination or dual-negative selection conjugation mutagenesis was used to generate *clpX* (VK055_2153), *clpP* (VK055_2154), and *clpXP* isogenic knockouts in TOP52 and 43816 as described (6, 56). Mutants were generated using primers described in Table S1. Mutants were confirmed by PCR amplification of the surrounding *clpXP* region and whole-genome sequencing. To generate the p*fimB* plasmid, primers were used to amplify *fimB* from genomic TOP52. PCR product and pBAD33 were double digested with XbaI and KpnI followed by ligation and transformation into TOP10 *E. coli* cells. The resulting plasmid was then electroporated into TOP52.

### Growth kinetics

Overnight cultures of each hvKp and cKp mutant were normalized to a standard OD_600_ and diluted 1:1,000 in LB. 100 µL of each strain was loaded in replicates of 10 into a 96-well plate and grown shaking overnight at 37°C with OD_600_ measurements taken in 15 min intervals (BioTek Synergy neo2).

### Mucoviscosity assay

Mucoviscosity was quantified as previously described (6). Briefly, overnight cultures were centrifuged at 9,400 × *g* before decanting the supernatant and resuspending the pellet in PBS to an OD_600_ of 1.0. The bacterial suspensions were then centrifuged at 1,000 × *g* for 5 min before the OD_600_ of the supernatant was measured. Each sample was tested in triplicate.

### Antimicrobial susceptibility assay

Three milliliters of LB was inoculated with the desired strain and grown shaking at 37°C until an OD_600_ of 0.1 was reached and then diluted in LB to create a standardized inoculum. In a 96-well plate, 50 µL of sterile LB was added to column 1-10, with 100 µL of LB added to column 12 as a sterile control. One hundred microliters of a 128 µg/mL solution of the desired antibiotic was added to column 11 and serially diluted to column 2 before 50 µL of the standardized inoculum was added to columns 1-11. The plate was then covered with a Breathe-easy membrane (Sigma-Aldrich) and grown at 37°C for 16 h before the OD_600_ values were measured. MIC was determined by the lowest concentration with no visible growth. Each strain was assessed in duplicate.

### Capsule quantification

The quantification of capsule was performed using a modified glucuronic acid assay (57). Briefly, bacteria were cultured in LB overnight and sub-cultured to OD_600_ of 0.2 and grown for 6 h at 37°C. Five hundred microliters of bacterial culture was mixed with Zwittergent 3–14 detergent to extract glucuronic acid and then precipitated with cold ethanol at 4°C for 1 h. The precipitate was then dried, resuspended in water, and dissolved by boiling with 12.5 mM tetraborate in concentrated H_2_SO_4_. After cooling, samples were incubated with a 3-hydroxydiphenol and the OD_520_ of each sample was measured against a standard curve of glucuronic acid (Sigma-Aldrich).

### Capsule locus promoter fusions

To determine expression of capsule genes, pPROBE-tagless, pPROBE-*manC*, pPROBE-*galF*, or pPROBE-*wzi* was transformed into wild-type, *∆clpX, ∆clpP*, and *∆clpXP* mutants in the TOP52 and 43816 backgrounds. Each strain was grown statically overnight at 37°C in LB-KAN (50 µg/mL) and sub-cultured to an OD_600_ of 0.2, diluted 1:100 in LB-KAN and grown at 37°C for 6 h. The cultures were washed twice in PBS and diluted 1:10. One hundred microliters of diluted culture was loaded in quadruplicate into a black 96-well plate. The relative fluorescent units (RFUs) were measured using a plate reader and normalized to the OD_600_.

### Cell culture

Human lung epithelial A549s (ATCC, CCL-185) were grown in Dulbecco’s modified Eagle medium (DMEM; Gibco Life Technologies) supplemented with 10% heat-inactivated fetal bovine serum (FBS) and 1% penicillin/streptomycin in 75 cm^2^ cell culture flasks (Gibco). Cells were grown at 37°C in a humidified 5% CO_2_ atmosphere.

### Epithelial infection assay

A modified macrophage infection assay was adapted to A549 cells and used to assess the invasion and adhesion of lung epithelial cells by selected strains (58). A549 cells were seeded into 24-well plates at a density of 2 × 10^5^ cells per well 48 h prior to infection. Overnight cultures of bacteria were collected, washed with PBS, and diluted in DMEM to achieve a multiplicity of infection (MOI) of 10. Prior to infection, the growth media was aspirated and washed twice with PBS before 0.5 mL of bacterial inoculum was added. Plates were centrifuged at 1,000 rpm for 10 min and incubated at 37°C for 1 h. The media was then aspirated and washed with PBS. The invasion plates then received DMEM media supplemented with 30 µg/mL gentamicin, while the adhesion plates received DMEM media. Plates were incubated for 1 h before the media was replaced with DMEM media supplemented with 5 µg/mL gentamicin for the invasion plates and DMEM media for the adhesion plates. Plates were incubated for 2 h and washed twice with PBS, and the cells of half the infected wells were lysed with 0.5% saponin. The remaining wells were used for a MTT cell viability assay. Serial dilutions of the lysate were plated onto LB agar and grown at 37°C overnight to determine bacterial count. Bacterial adherence was determined by calculating the difference between the adherence and invasion bacterial count. Bacterial adherence and invasion CFUs were normalized to cell viability and the initial inoculum.

### Cell viability assay

Three hundred microliters of PBS and 30 µL of a 5 mg/mL MTT (3-(4,5-dimethylthiazol-2-yl)−2,5-diphenyltetrazolium bromide, ThermoFisher) PBS solution was added to each well. Plates were incubated for 4 h at 37°C before 300 µL of a 0.01 M HCl-SDS solution was added to each well. Plates were incubated for 16 h before measuring the OD_570_.

### Phase assay for type 1 pili

A PCR-based assay was used to determine the orientation of the *fimS* phase switch based on a previously established method (28, 59). Briefly, PCR primers (Table S1) were used to amplify an 816 bp region of *fimS*. The PCR conditions were as follows: one cycle of 94°C for 1 min; 40 cycles of 94°C for 1 min, 58°C for 70 s, 72°C for 70 s, and one cycle of 72°C for 3 min. The amplified PCR product was then digested with HinfI (New England BioLabs) for 2 h at 37°C. The products and a 100 bp DNA ladder were resolved on a 2% agarose gel stained with SYBR Safe (0.5 µg/mL) in 1× Tris-Acetate-EDTA (TAE) at 150 V for 45 min and visualized with UV light. A phase-ON switch produced amplicons of 605 bp and 212 bp, while a phase-OFF switch produced amplicons of 496 bp and 321 bp.

### Immunoblotting

Acid-treated whole cell lysates were prepared as previously described and separated by SDS-PAGE (60). Gels were transferred to a 0.2 µm PVDF membrane and blocked with 3% nonfat milk and 2% bovine serum albumin in PBS with 0.1% Tween-20. Immunoblotting for FimA used 1:2,000 rabbit anti-type 1 pilus primary antibodies (Biosynth) and immunoblotting of MrkA used 1:10,000 chicken anti-MrkA primary antibodies (Biosynth). RNA Polymerase subunit α (RpoA) was used as a protein loading control (1:2,000, Biolegend 663104). Blots were stained with 1:10,000 goat anti-rabbit IgG HRP for FimA, 1:10,000 rabbit anti-chicken IgY HRP for MrkA, and 1:5,000 sheep anti-mouse for RpoA. Immunoreactive bands were visualized with the Clarity enhanced chemiluminescence (ECL) substrate (Bio-Rad). Band intensities were quantified using ImageJ software (https://imagej.net/ij/) and normalized to the relative RpoA loading control.

### Mouse infections and organ titers

Female CD-1 mice aged 7–8 weeks were infected using non-surgical oropharyngeal aspiration as described in Wasbotten et al (61). Given the difference in size and ID_50_ of *K. pneumoniae* across mouse genders, exclusively female mice were utilized for consistent comparison across experiments. Briefly, mice were anesthetized with inhaled isoflurane and suspended by the upper incisors. The tongue was retracted laterally, and 50 µL of inoculum was administered to the oropharynx to be aspirated on subsequent breaths. Blood was collected 24 hpi via cardiac puncture into tubes containing 5 µL of sterile filtered 0.5 M EDTA. For organ titers, the lungs and spleen were harvested and placed into 900 µL of PBS and homogenized. The blood and organ homogenates were serially diluted, plated onto LB agar, and incubated overnight at 37°C to determine bacterial titers.

### Data analysis and statistical methods

Figures were created with GraphPad Prism (v 10.2) unless otherwise noted. Comparisons between two groups of continuous variables were analyzed using the Mann-Whitney *U* test followed by the Holm–Šidák correction for multiple comparisons, as not all data were normally distributed. All tests were two tailed with a *P* < 0.05 considered significant.

## SUPPLEMENTAL MATERIAL

Supplemental material iai.00680-25-s0001.pdf Fig. S1; Table S1.

ASM does not own the copyrights to Supplemental Material that may be linked to, or accessed through, an article. The authors have granted ASM a non-exclusive, world-wide license to publish the Supplemental Material files. Please contact the corresponding author directly for reuse.
