# Supplementary material for: Differential contributions of ClpX and ClpP to pulmonary virulence in classical and hypervirulent Klebsiella pneumoniae
Source: Infect Immun. 2026 Jan 30;94(3):e00680-25. doi: 10.1128/iai.00680-25 (PMC12974122; doi:10.1128/iai.00680-25)
Supplement: Supplemental material — Fig. S1; Table S1. [file iai.00680-25-s0001.pdf]

Supplemental Material

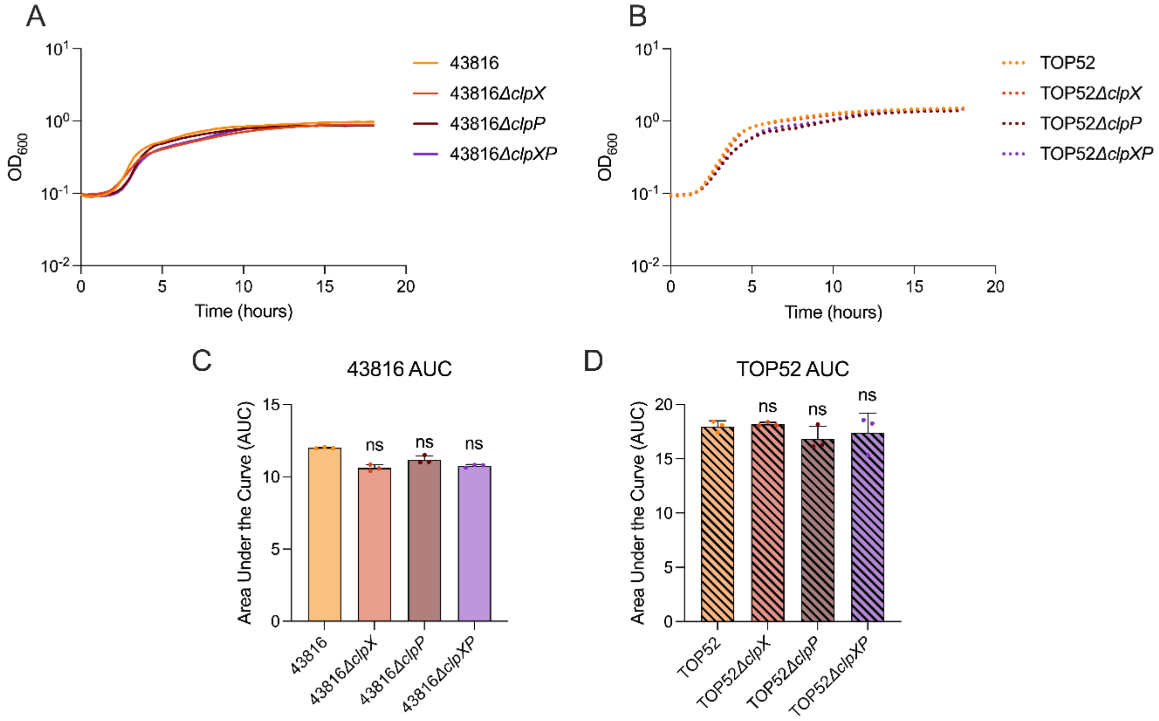

Supplemental Figure 1: Growth kinetics of wild-type and mutant strains of 43816 (A) and TOP52 (B). The OD_{600} was evaluated at 15 min intervals over a period of 18 h with samples done in triplicate. An area under the curve (AUC) analysis was performed for the 43816 (C) and TOP52 (D) backgrounds to determine the presence of growth defects. Significance was determined through comparison to the wild-type background by the Mann-Whitney *U* test with Holm-Šidák correction. ns, not significant.

Supplemental Table 1: Primer sequences used in this study.

**Primer Name**	**Use**	**Primer Sequence 5' → 3'**
ClpX-P1-F	Amplified kanamycin resistance cassette from pKD4 for insertion into *clpX*	tcatgacagataaacgcaaagatggatcgggc aaattgtt
ClpX-P2-R	Amplified kanamycin resistance cassette from pKD4 for insertion into *clpX*	gaaatggttaattattcaccagatgcctgctgagc ttccg
ClpP-P1-F	Amplified kanamycin resistance cassette from pKD4 for insertion into *clpP*	gagacaactttgcaccccatatggcccgtgtagg ctggagctgcttc
ClpP-P2-R	Amplified kanamycin resistance cassette from pKD4 for insertion into *clpP*	gcggcacccttgcgccttgggcatcaattacgat gggtcacatatgaatatcctccttag
ClpXP-P1-F	Amplified kanamycin resistance cassette from pKD4 for insertion into *clpXP*	gagacaactttgcaccccatatggcccgtgtagg ctggagctgcttc
ClpXP-P2-R	Amplified kanamycin resistance cassette from pKD4 for insertion into *clpXP*	gaaatggttaattattcaccagatgcctgctgagc ttccg
ClpX Check F	Verifies Δ*clpX* sequence	gggtgccgctttacgcttc
ClpX Check R	Verifies Δ*clpX* sequence	ccacactccactgcatcgtc
ClpP Check F	Verifies Δ*clpP* sequence	gaaactgtggctgataatccgtccg
ClpP Check R	Verifies Δ*clpP* sequence	gcccttcattagtatatacacaaaatccttcgc
ClpXP Check F	Verifies Δ*clpXP* sequence	gaaactgtggctgataatccgtccg
ClpXP Check R	Verifies Δ*clpXP* sequence	ccacactccactgcatcgtc
ClpX Up F	Amplification of upstream region of *clpX* for homologous recombination	gacggtatcgataagcttgatatcggcgcagatg ctgtttctggaagcgg
ClpX Up R	Amplification of upstream region of *clpX* for homologous recombination	gtgaaatggttaagagtccaaacctctttttaagtt ctttgtgccg
ClpX Down F	Amplification of downstream region of *clpX* for homologous recombination	aggtttggactcttaaccatttcactcaagcagtta acaaaaagggggattttatctcc
ClpX Down R	Amplification of downstream region of *clpX* for homologous recombination	actaaagggaacaaaagctggagcttgtgcgc cgcaatggtatccgccag
ClpP Up F	Amplification of upstream region of *clpP* for homologous recombination	atcgataagcttgatatcgtcaggctgctcagcgt ttcg
ClpP Up R	Amplification of upstream region of *clpP* for homologous recombination	ttgggcaatggggtgcaaagttgtctcg
ClpP Down F	Amplification of downstream region of *clpP* for homologous recombination	caccccattgcccaaggcgcaagggtgc
ClpP Down R	Amplification of downstream region of *clpP* for homologous recombination	gaacaaaagctggagctttcggtcagggtggtg gcgtc
Phase A	Amplifies *fimS* for switch orientation analysis	gggacagatacgcgtttgat
Phase B	Amplifies *fimS* for switch orientation analysis	ggcctaactgaacggtttga
XbaI-FimB-F	For cloning *fimB* into pBAD33	ctaa tctaga atgcctgcaataaaagggaaaacc
KpnI-FimB-F	For cloning *fimB* into pBAD33	ctaa ggtacc atgcctgcaataaaagggaaaacc
